# A Grain of Wheat from a Bushel of Chaff

**Published:** 1859-03

**Authors:** 


### *A Grain of Wheat from a Bushel of Chaff.*

From that eventful morning when the infant Cain, playing at his mother's knee, proclaimed by 'raising his voice in tuneful song' an important discovery by the simple experiment of bringing his little nose in violent contact with the ground, to the day when Pat Terrifer, with his nether limb mashed beyond all hopes of redemption, lay meditating on his narrow hospital-pallet upon the dubious means of relief promised him by the attending surgeon, there has been a steady but unequal contest by man against the curse imposed upon him for the first fault—a constant endeavor to strike the word 'pain' from the vocabulary.

And yet, although ages have rolled on, and millions upon millions have lived, pondered, experimented, and suffered, so meagre was the result, that even so late as 1839 the celebrated French surgeon Velpeau, despairing of any solution to the problem, declared that 'to escape pain is a chimera which we are not permitted to look for in our day; that the cutting instrument and pain in operative medicine are two words which never present themselves the one without the other.' And yet he has lived to see his opinion changed. Only seven years after, as one of a commission appointed to examine the merits of a new means of relief proposed, he heard and acquiesced in the glad announcement made to the whole world: 'We have conquered pain.'

How was this glorious victory gained? Who won it? Was it a mere spontaneous, suddenly-imagined suggestion which luckily found a coroborative solution at once in experiment? Or was it a result from a series of trials and failures—a fact settled by a slw process of reasoning on a certain amount of given information ?

Such are the questions often proposed—let us see if all of them can be answered satisfactorily. One, certainly the last, can be at once replied to affirmatively, for the facts to prove it are sufficiently numerous and well substantiated to satisfy even the most obdurate Mr. Gradgrind. Our object must be therefore to see at what period the first attempts were made to relieve pain, and in what they consisted.

Throwing aside, as doubtful, the story of the sleepy action of the nepenthe upon Ulysses and his companions; disputing with the Biblical commentators the theory that narcotics were given to the unfortunates about to be crucified; disbelieving entirely the assertion of Herodotus that a narcotic intoxication was common in his day; we reach at last a tangible fact and fixed date.

A worthy gentleman of Naples, by the name of Pliny, who, it is known, was living in the first years of our era, has written us a letter in most excellent Latin, in which he declares that it was the custom to give a certain decoction of herbs 'before cuttings and puncturings, lest they should be felt.' One would suppose this so satisfactory, that no doubt could be harbored as regards his integrity. But apparently not satisfied with the probable effect of so simple an assertion, he instantly proceeds to perpetrate a most abominable Munchausenism about a stone which he calls Memphites, declaring that it will produce the same effect. But even this admits of some explanation, for, as by his direction, it was necessary to apply it in order to stupefy the part, it is highly probable it was to be used as Mr. Montgomery intended to use his brick. That omnipresent race, however, the Chinese, who seem inclined to cheat us out of all claim to priority of invention, have taken issue with all the rest of the world, by declaring that from the remotest ages it has been the custom of their medical men to give patients a narcotic powder, so that no pain need be felt. In a curious book in the Imperial Library at Paris, called Kou-kin-i-tong, we find the name Ma-yo given to this powder, which was probably no more than the Indian hemp now so extensively used throughout the East, under the name of Bhang, to produce a temporary intoxication, and the same drug which in the form of an extract is the bane of the hashheesh-eater.

Constantly through the years succeeding the death of Pliny, from Dioscorides, from Matthiolus, from the spirited narrative of Marco Polo, and all the chroniclers of the crusades, from the old historians of the East, from the records of the Inquisition, and the published cases of the long series of illustrious surgeons down to this very year, we find mention made of attempts to relieve pain, while equally often proof is given of their inefficiency and failure.

The reason of this is obvious: with few exceptions, their experiments were directed toward the effects of solid narcotic substances upon the system. It was the substances used, and not the method, which caused the failures. Opium, which is one and perhaps the best of all narcotics, if given in sufficient amount to wholly deaden pain (which can be done,) possesses the most disagreeable property of deadening the recipient so utterly, that he rises no more in this world in a bodily form; consequently the user is always placed in the disagreeable dilemma of inflicting a certain amount of pain and keeping on the safe side, or of risking a coroner's inquest and a verdict of manslaughter and malpractice. The trials, however, demonstrated one valuable fact, that as when swallowed they produced slowly a much more continued and excessive stupefaction than was needed, it was important to substitute some article which should produce the effect more quickly—more safely, even it were used a little carelessly; and above all, one which should not cause a persistent condition when the administration had been stopped.

This was the first step toward our present state of knowledge. When the men of science had arrived at this conclusion, they all fell to experimenting and suggesting year after year. Old Baptista Porta proposed what he called his sleeping-apple, 'the smelling of which binds the eyes with a deepe sleepe,' which was a ball formed of some narcotic drugs, that was to be kept from the air, and when wanted for use, was to be held under the nose. Cold was another means, as it was found that when a man was nearly frozen to death, his sensibility to pain was much blunted. Pressing upon the nerves until there was no sensation in the limb; choking and bleeding the patient until he was all but insensible, were other plans. Mesmer advanced the theory of animal magnetism, and made many converts; but whether before or after they were operated upon, has never been decided.—Finally, some ingenious man, whose name is wholly unknown to fame, suggested the use of alcohol—that is, the patient should be made so drunk that he could feel nothing. This being a pleasant form, met with much success, and was the second step onward. But still with this there were some faults. It was found that it required too long to produce the effect, that it was not caused equally in different persons, that it asted too long, and lastly, that it was somewhat expensive and dangerous. So the wits of the chemists were set to work to devise something better than alcohol.

There is now existing near Naples, and records concerning it date back as far as Pliny, a cave called 'Grotta del Cane,' which is probably one of the outlets through the volcanic crust of which the whole vicinity of Vesuvius is composed, as from it constantly exhales a steam which is found to contain large quantities of carbonic-acid gas. This gas, which is heavier than common air, is totally destructive to animal life, if sufficient time be given for its effects; and any animal placed in it will die, unless quickly removed from it into the pure air.—Taking advantage of this known fact, it was proposed to administer it in such quantity as to produce its insensible but not deadly effect, and that instantly on its appearance the administration should be suspended, and the operation be performed during the interval before perfect recovery.

This was the third step. But unfortunately its use was found to be too dangerous; and although it was advocated by intelligent men, and it was stated that some operations were performed under its influence, it fell into disfavor, and has never since been resuscitated. But it gave the right direction to investigation. In 1799 Humphry Davy, (not then Sir) who was an assistant in an institution in England for the treatment of disease by the inhalation of various substances, commenced a series of experiments with nitrous oxyde, or as it is often called, laughing-gas. These he published, and although he does not seem to have rendered himself by its use, at any time, wholly insensible he must have caused some decided effect, for he has written; 'As it appears capable of destroying physical pains, it may probably be used with advantage during surgical operations.' This was the fourth important step.

But his was only a suggestion—a proposition which was never put by him to the test of experimentation: its death was coïncident with its birth as far as any real benefit accrued from it to mankind. Nearly fifty years afterward, in the winter of 1844, a public lecture was given in the city of Hartford to illustrate the effects of this very agent, the laughing-gas. Among the audience was a person by the name of Horace Wells, who struck by its effects upon one of the persons who had inhaled it, made the causual remark: 'That he believed that a person (under its influence) could undergo a severe surgical operation without feeling any pain.' He offered to inhale the gas himself and allow one of his teeth to be extracted. He did inhale it that very evening: a tooth was extracted, and, as he asserted, without the slightest sensation of pain. This was the fifth great step, the demonstration of an invaluable principle—in fact the discovery.

A number of experiments were subsequently made with the gas both by himself and others, and as is shown by the affidavits of many good and reliable men, with an eminent degree of success; for many dangerous and ordinarily painful operations were performed upon persons who took oath they had experienced no pain whatever. But two objections were found with it: its preparation was some what troublesome, and it was rather too bulky for transportation. He accordingly searched for some other agent which should more fully and perfectly fill the needful indications. There was then sold in every druggist's shop an article of common use in medicine, the effects and method of managing of which, had been perfectly well known for over five hundred years. This medicine was called an ether, the name of the chemical acid used in its manufacture being prefixed to it by way of designation. As there are many kinds of acids, there are consequently many kinds of ethers; of these the most common is sulphuric ether, sulphuric acid being used in its fabrication. It is a clear liquid, like water, highly volatile, intoxicating in its effect, but in a much more rapid and excessive degree than alcohol. For over fifty years it had been recommended by medical men for inhalation in certain diseases of the lungs; and from individual cases where it had been used in this way, it was known that a certain amount of intoxicating effect would be produced. Reasoning on the fact which had been before experimented upon, that alcohol would produce a suspension of personal suffering, and the similarity of effect known to be ceused by the inhalation of ether, Mr. Wells determined to try if it would supply the deficiency. This was the sixth, and the step which has left us at our present state of knowledge.

But although much was anticipated by him from the ether, he did not consider it wholly satisfactory, for he ultimately returned to the use of his first agent, the nitrous oxyde gas. During a visit made by him to Boston that same winter, he communicated his discovery to an old friend and partner, named Morton. On the sixteenth day of October, 1846, this same Morton made his appearance at the Massachusetts General Hospital in Boston, and there, in the presence of a large number, of surgeons and spectators administered this ether to a man from whom was then removed a large tumor, without his having perienced the slightest pain. As from that day the surgeon has been able to gauge the amount of suffering he will inflict in any operation as accurately as the corner-grocer can weigh out a pound of sugar; as narcotics, for three thousand years the sole champions against inflicted pain, unconditionally vacated the arena on the approach of the new-comer, it would seem the easiest thing in the world to tell the time of the discovery, and the name of the man who really conferred it.

Three men have stood before the world as claimants for the honor. Horace Wells for his acknowledged use of nitrous oxyde gas in 1844; William T. G. Morton upon the undisputed ground of his public exhibition in 1846; and lastly, Dr Charles T. Jackson, who makes the positive personal assertions that he, in 1842, by the accident of inhaling an excessive amount of ether, made the discovery that it would produce a perfect insensibility, and that it was from his information, and at his instigation, that Morton performed the conclusive experiment at the hospital.

By priority of date, it is obvious that the credit should be awarded Dr. Jackson, provided it were perfectly proved, first, that he did discover in 1842 what he asserts, and second, that he reduced what was at first but a theory to a certainty, by the test of actual experiment.—But here is the dilemma. Nothing was heard of his discovery and claim, until subsequent to its verification by another, while out of his own mouth, it is proved that he never experimented upon what he considered so invaluable a discovery. Whether he induced another to experiment for him, is a simple question of veracity, in which the public have little interest; but as he kept his secret so well for four years, it is allowable to suppose that humanity might have been none the wiser at the end of forty.

Morton, who evidently considers the pen as mightier than the sword and makes up by multiplicity of documents for weakness of proof, makes a direct denial that two years before his public appearance, he knew that Wells was experimenting with nitrous oxyde, and that he conversed with him concerning it. Some corroborative testimony is evidently needed to show when he formed and experimented upon the theory. The claim of Horace Wells rest upon testimony showing, that from 1844 to 1846, he used both ether and nitrous oxyde gas, to produce anæsthesia; upon testimony showing that he communicated his knowledge directly to Morton, and probably indirectly to Jackson. Could more be required to establish any demand? Should not this grain of truth, picked from the bushel of chaff with which the antagonism of others has enveloped it, be sufficient, under the benign influences of honest investigation, to produce a harvest of honor to the memory of a man, who died unnoticed and unrewarded, after bestowing one of the greatest blessings ever conferred on suffering man?

[*Knickerbocker.*]

